# Reliability of Semiautomated Computational Methods for Estimating Tibiofemoral Contact Stress in the Multicenter Osteoarthritis Study

**DOI:** 10.1155/2012/767469

**Published:** 2012-10-14

**Authors:** Donald D. Anderson, Neil A. Segal, Andrew M. Kern, Michael C. Nevitt, James C. Torner, John A. Lynch

**Affiliations:** ^1^Department of Orthopaedics and Rehabilitation, The University of Iowa, Iowa City, IA 52242-1088, USA; ^2^Department of Biomedical Engineering, The University of Iowa, 1402 Seamans Center, Iowa City, IA 52242, USA; ^3^Department of Epidemiology, The University of Iowa, 2181 Westlawn, Iowa City, IA 52242-1088, USA; ^4^Department of Radiology, The University of Iowa, Iowa City, IA 52242-1088, USA; ^5^Department of Epidemiology and Biostatistics, University of California, San Francisco, 185 Berry Street, Lobby 5, Suite 5700, San Francisco, CA 94107-1762, USA; ^6^Department of Radiology, University of California, San Francisco, 185 Berry Street, Lobby 5, Suite 5700, San Francisco, CA 94107-1762, USA

## Abstract

Recent findings suggest that contact stress is a potent predictor of subsequent symptomatic osteoarthritis development in the knee. However, much larger numbers of knees (likely on the order of hundreds, if not thousands) need to be reliably analyzed to achieve the statistical power necessary to clarify this relationship. This study assessed the reliability of new semiautomated computational methods for estimating contact stress in knees from large population-based cohorts. Ten knees of subjects from the Multicenter Osteoarthritis Study were included. Bone surfaces were manually segmented from sequential 1.0 Tesla magnetic resonance imaging slices by three individuals on two nonconsecutive days. Four individuals then registered the resulting bone surfaces to corresponding bone edges on weight-bearing radiographs, using a semi-automated algorithm. Discrete element analysis methods were used to estimate contact stress distributions for each knee. Segmentation and registration reliabilities (day-to-day and interrater) for peak and mean medial and lateral tibiofemoral contact stress were assessed with Shrout-Fleiss intraclass correlation coefficients (ICCs). The segmentation and registration steps of the modeling approach were found to have excellent day-to-day (ICC 0.93–0.99) and good inter-rater reliability (0.84–0.97). This approach for estimating compartment-specific tibiofemoral contact stress appears to be sufficiently reliable for use in large population-based cohorts.

## 1. Introduction

Osteoarthritis (OA) is the most common chronic joint disease [1, 2] and a major cause of disability in older adults [3]. In the United States, more than 21 million individuals, 16% of the population, suffer from arthritis. This estimate is expected to reach 18.2% by 2020, affecting nearly 60 million Americans [2, 4]. Most importantly, pain associated with knee OA contributes to substantial functional limitations and disability [5], prompting many individuals to seek frequent medical care [2, 6]. Epidemiological risk factors such as increased malalignment and obesity are well-established risk factors for development of knee OA. However, these factors only indirectly reflect aberrant joint mechanics, and they cannot account for local site-specific biomechanical factors involved in OA pathogenesis.

In contrast, articular contact stress is a critical and direct factor in joint health. The addition of a valid biomechanical model of local contact stress would therefore enhance the ability of an epidemiological model to predict incident symptomatic tibiofemoral OA and anatomic worsening, by identifying both the individuals and the area of the joint surface at greatest risk. Ideally, any such subject-specific modeling approach would be suitable for use in large population-based study cohorts.

Contact stress is of course only one quantitative measure of the full (multiaxial) stress state to which cartilage is subjected. But, in addition to having been shown to be a very useful metric in its own right [7, 8], it also closely correlates with shear stress at the osteochondral junction and with other important stress measures under functional loading [9].

Prior computational methods to determine articular contact stress have primarily relied on finite element analysis (FEA). However, FEA is less than ideally suited for population-based longitudinal studies, due to its relatively high complexity and resource-intensive nature for handling nonlinearities inherent in the modeling of multibody contact. Fortunately, discrete element analysis (DEA) has emerged as an expeditious alternative to FEA for determining articular contact stress [10, 11]. In DEA, bones are treated as rigid bodies and cartilage as compressive-only springs, distributed over the articulating bone surfaces. DEA modeling approaches have begun to be developed to estimate joint contact stress in a subject-specific manner [12–14]. But, analyses using these methods have been limited to tens of subjects, due to inherent complexities in subject-specific modeling. Much larger numbers (likely hundreds, if not thousands) of subjects will need to be analyzed to achieve the statistical power necessary to clarify the role of contact stress in joint pathology.

The Multicenter Osteoarthritis (MOST) Study is a prospective observational study of adults, age 50–79 years at baseline, with either preexisting knee OA or at elevated risk for it based on frequent knee symptoms, history of knee injury, or surgery, being overweight, or obese [15]. Large-scale studies, such as MOST, present a unique opportunity to use longitudinal medical imaging data (CT, MRI, radiographs) to assess the relationship between articular contact stress and pathology. Using a DEA methodology, we have previously shown that baseline articular contact stress can predict incident symptomatic knee OA development over 15 months in MOST study participants [16]. We have furthermore also demonstrated that elevated articular contact stress can predict the risk of cartilage and bone marrow lesion worsening over 30 months in subjects from MOST [17]. Our initial study involved modeling and analysis of 60 knees, and the subsequent study an additional 38 knees. While these studies have begun to shed new light on the pathomechanical origins of OA, again, greater understanding will require analysis in much larger numbers of subjects.

This study assessed the reliability of semiautomated modeling and analysis methods developed to estimate articular contact stress using data from the MOST cohort. We have previously validated our DEA-based methods by demonstrating good agreement between estimated contact stress values and those measured in cadaveric specimens [12]. Prior to use of DEA to estimate tibiofemoral contact stress in larger observational or interventional studies, it is also important to determine the interrater and day-to-day reliability of MRI segmentation and of model registration methodologies. If found to be a reliable approach, DEA-based contact stress assessment could be a viable technique for efficient collection of longitudinal tibiofemoral contact stress in large-scale studies of the role of biomechanical risk factors in the pathomechanical origins of knee OA.

## 2. Materials and Methods

This study was conducted using 10 knees randomly selected from a prior study nested within the MOST cohort of 3,026 adults with or at elevated risk for knee OA [16]. MOST used a population-based sampling frame to recruit 3,026 community-dwelling men and women, age 50–79 years, with frequent knee symptoms or at risk for developing symptomatic knee OA based on a history of knee injury or surgery or being overweight or obese. Exclusion criteria included bilateral knee replacement, cancer, or rheumatologic disease. For the prior nested study, 30 case knees were randomly selected from among MOST subjects at one clinical site who developed incident symptomatic tibiofemoral OA between their baseline and 15-month follow-up visits. These case knees were matched with knees from 30 control subjects, randomly selected from the same cohort, followed over the same time, at the same site, who did not develop the combination of frequent knee symptoms and radiographic tibiofemoral OA.

### 2.1. Mechanical Modeling Approach

Accurate mechanical modeling required that 3D bone surfaces be segmented from MRI images and aligned to a loaded apposition. Posterior-anterior (PA) fixed-flexion weight-bearing knee radiographs, acquired according to a standardized protocol [18], were used in a 3D-to-2D registration of bone surfaces to a loaded apposition [19–22]. A feature-based 3D-to-2D alignment algorithm was written in MATLAB (The MathWorks, Natick, MA). The algorithm required a 3D triangulated surface for each bone (available from the MRI segmentations), as well as a 2D weight-bearing radiograph with a binary tracing of the relevant bone edges.

**Figure 1:**
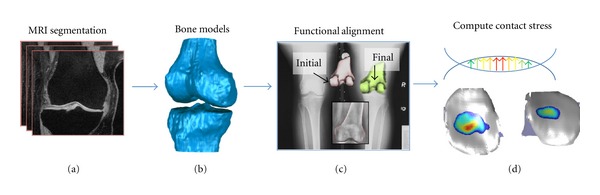
Methodology for subject-specific, population-wide investigations of habitual contact stress exposure in the knee. MR images are segmented to produce bone models (a and b), which are aligned to a standing radiograph using a ray casting algorithm (c). Contact stress is computed using discrete element analysis (d).

The bony geometry of each knee was obtained from the baseline visit MRI. MR images were acquired in MOST with the subject in a seated position, using a 1.0 T dedicated knee system (ONI Medical Systems, OrthOne). DEA knee model generation involved several steps (Figure 1). First, bone surfaces of the tibia and femur were segmented slice-by-slice from MR images by tracing boundaries using an interactive pen display [23]. The tracing was done within the OsiriX DICOM viewer software environment (OsiriX Foundation, Geneva, Switzerland). Point clouds for each traced bone were output and wrapped with surface triangulations using Geomagic Studio software (Geomagic, Inc., Research Triangle Park, NC). Moro-oka et al. previously showed that manual MRI segmentation could provide model surfaces for the femur and tibia that differed from CT-based segmentations by only 0.08 mm and 0.14 mm, respectively [24]. Any hole in the surface triangulation was filled using local curvature-matching methods. Application scripting of the Studio software afforded an efficient means to produce these surfaces for a large number of segmented knees. Application scripting enables the repeated running of a program (in this case, Geomagic Studio) to produce a desired output (in this case, geometrical models) using a series of text-based commands. Scripting, thus, makes possible the unattended batch execution processing to create a large number of models.

The registration alignment procedure utilized an optimization approach to minimize the difference between the segmented bone model silhouette, as projected onto a pseudo-radiographic image plane using a ray-casting technique, and information from the PA fixed-flexion radiograph [19]. Bone edge tracings from the (2D) radiographs were obtained in a supervised manner using a semiautomated procedure implemented in MATLAB. This process enables a user to confirm that the edges identified by the semiautomated algorithm correspond with the bony outline and not to spurious trabecular detail or radiographic artifacts. Next, a scene was recreated in virtual space to match the MOST radiograph acquisition protocol, with the X-ray source placed at 72 inches from the detector and angled at a 5°, 10°, or 15° caudal angle, depending upon the details of the original radiographic acquisition. The radiographic detector was represented virtually as a rectangle constructed of two adjacent polygons located appropriately in the scene. The spatial coordinates of the segmented bone model were then transformed from the MRI coordinate space to a nominal position between the source and detector in the virtual scene.

Rays were cast from the X-ray source to the silhouette edges of the model and intersected with the film plane, creating a set of points that defined edge vertices projected onto the film. These projections were then connected using a line drawing algorithm to create a continuous contour representing the bone edge. This contour was compared to the bone edge tracings from the fixed flexion radiograph. Comparison between the ray-casted contour and the segmented radiographic edge provided a basis for a cost function to align the bone model.

**Figure 2:**
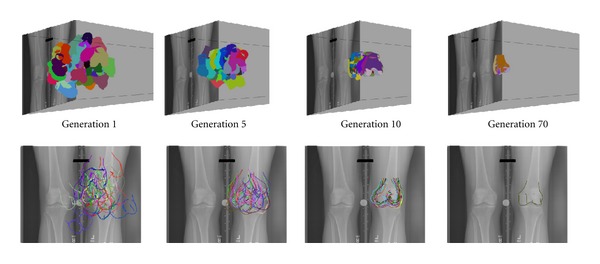
Multiple evolutionary generations in the CMA-ES optimization approach show a steady march toward a single best alignment (top row), driven by a cost function that incorporates evaluation of agreement between a model silhouette projected onto the radiographic image plane and the bone edge detected from the actual radiograph (bottom row). Each CMA-ES generation includes 70 candidate spatial alignments, with those having the lowest cost function evaluation being used to drive subsequent generations.

A covariance matrix adaptation evolution strategy (CMA-ES [25]), a meta-heuristic global optimizer that requires few parameters to be selected a priori, was utilized to iteratively manipulate bone models through the 3D space to achieve the desired best alignment (Figure 2). The translations and rotations necessary to bring the bones into loaded apposition are output from the software. As CMA-ES is a heuristic algorithm and this specific class of problem is highly nonconvex, three runs of the algorithm were executed, and the results were recorded. In recent work with a similar alignment approach, excellent measurement accuracy was established with translations accurate to within 0.5 mm and rotations to within 0.7° [21].

Each of the two bones (femur and tibia) was aligned separately, but not independently. First, the femur was aligned, and then the final best computed transformation for the femur was applied to the tibia to move it into an initial pose. An additional component was then added to the cost function to penalize for movement of the tibia away from the femur. Starting from this position of close approximation to the final best femoral alignment allowed for the tibial alignment to proceed much more expeditiously.

Following alignment, articular contact stresses were computed using a previously validated DEA algorithm, written in MATLAB [12]. The stress analysis assumed rigid subchondral bone, with a uniform combined tibiofemoral thickness 6 mm linear elastic cartilage layer. This assumption of uniform cartilage thickness was necessary due to scan resolution-related issues; precise measurement of the cartilage thickness was not feasible. Sensitivity to the combined cartilage thickness was minimal; trials with thickness of 4 and 8 mm showed <10% change in peak contact stress [12].

The DEA algorithm allows rapid computation of contact stress between two apposed surfaces, without the necessity for a volumetric meshing step as in finite element analysis. The algorithm begins by using a space-partitioning algorithm to expeditiously compute nearest neighbors between facets of the apposed surfaces. Each nearest neighbor pair was queried to identify pairs that had undergone apparent penetration and create springs between those pairs. Contact stresses were then computed using a spring model [13] that related deformation of the springs to engendered contact stress [12]. The computation was based on total cartilage thickness, the cartilage elastic modulus and Poisson's ratio (chosen to represent cartilage behavior at physiologically relevant loading rates), and the calculated spring deformations associated with the applied bone displacements. The elastic modulus of cartilage used was 12 MPa [26], and the Poisson's ratio was 0.42 [27].

The overall contact force was computed from the vectorial summation of normal forces acting on each individual triangle (contact stress × triangle area). The simulation was run in load control, utilizing a vertical loading of 1000 N. Based upon the computed contact force, the tibiofemoral apposition was adjusted in an iterative manner to obtain the translations required to achieve static equilibrium. The peak and mean contact stresses acting over each compartment (medial and lateral) of each knee were reported from the DEA-computed contact stress distributions.

### 2.2. Reliability of the Modeling Approach

To determine the reliability of our modeling approach, we assessed the reproducibility of DEA contact stress measures obtained from (1) multiple independent segmentations of ten MOST participant knees based on a single set of registrations as well as (2) multiple independent registrations based on a single set of segmentations, as described in detail below.

To assess reliability of the segmentation step, three individuals manually segmented bone surfaces from sequential MRI slices on two nonconsecutive days. Each independent segmentation was then subjected to the same spatial matrix transformation for placement into a loaded apposition. DEA methods were used to estimate contact stress distributions for each knee. Reliability of the segmentation step (day-to-day and interrater) was assessed by calculating intraclass correlation coefficients (ICC 2,1) for peak and mean tibiofemoral contact stress using the Shrout-Fleiss single-score method [28].

To assess reliability of the registration step, four individuals registered the 3D models based on a single set of bone surface segmentations (i.e., all individuals used the same segmentations) of the same 10 MOST knees to weight bearing radiographs. One individual repeated the task at a later point in time. The registration methods described above were used to align the MRI-derived 3D bone models to the bone edges from the radiographs. Thus, the registration reliability summarized the agreement between contact stress estimates, considering the aggregate variability from users interactively selecting the relevant radiographic bone edges and variability due to differences in the outcomes of the three runs of the CMA-ES alignment algorithm. Reliability of the registration step (day-to-day and interrater) was assessed by calculating intraclass correlation coefficients (ICC 2,1) for peak and mean tibiofemoral contact stress using the Shrout-Fleiss single-score method [28].

## 3. Results and Discussion

The methods described provided an efficient means for obtaining contact stress estimates in the knees studied. Manual tracing of the bone surfaces accounted for the majority of time expenditure, at approximately 2 hours of user time per knee. Suitable automated knee segmentation methods (e.g., [29]) are fast becoming available, and they will allow for large reductions in the user time required to complete this task.

Alignments were completed in approximately 4 minutes per bone, involving over 8,000 cost function evaluations. As expected, alignment had the highest variability out of the plane of the radiograph. Future studies using simultaneous biplanar imaging techniques would greatly reduce this variability, but PA and lateral images were unfortunately acquired asynchronously in MOST. Contact stress computations were completed in approximately 3 minutes per knee and produced reasonable contact stress distributions, consistent with those reported in our prior work with this modeling approach [16, 17].

**Figure 3:**
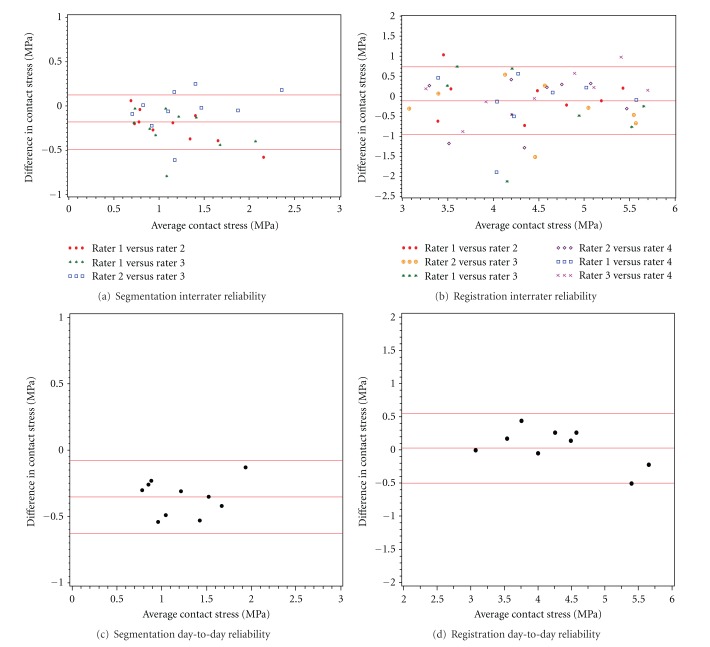
These Bland-Altman plots show day-to-day and interrater reliability for the segmentation and registration to generate estimates of the mean contact stress on the medial compartment. For day-to-day reliability, the *y*-axis represents the difference between measurements on two separate occasions for a single rater, while the *x*-axis represents the mean value. For interrater reliability, the *y*-axis represents the difference between measurements obtained by two different raters while the *x*-axis represents a pooled mean of all rater pairs. For all plots, the central horizontal line is the mean difference and the lines above and below it represent ± 2 SD. Measurements that resulted in values of zero (no contact stress) were omitted from the final plots to enhance legibility.

The (day-to-day, interrater) reliabilities for segmentations of the medial compartment were (0.94, 0.87) for peak and (0.93, 0.84) for mean contact stress, and for the lateral compartment they were (0.99, 0.96) and (0.98, 0.95), respectively (Table 1). The (day-to-day, interrater) reliability of the registrations for the medial compartment were (0.93, 0.94) for peak and (0.94, 0.95) for mean contact stress, and for the lateral compartment they were (0.95, 0.97) and (0.96, 0.97), respectively (Table 2). Bland-Altman plots of day-to-day and interrater reliability were generated for visual assessment of the reliability of segmentation and registration steps in the estimation of the peak and mean contact stress on the medial and lateral compartments. The four plots for medial compartment mean contact stress, a key predictor of the development of knee OA in our prior work [16, 17], are presented in Figure 3.

The quality of the contact stress estimations obtained using the methods here presented ultimately depends on the input data, and that is why reliability assessments were undertaken. Alignment is a major issue, but sotoois the reliability of the manual segmentations. Both significantly influence the computed values of contact stress, so their reliabilities were independently assessed. The variability in contact stress calculations associated with different segmentations and registrations were evaluated precisely because that computation produces the primary variable demonstrated to have clinical predictive value in our other published work—where the rubber hits the road in efforts to correlate estimates of contact stress with the development of knee OA.

Computational stress analysis necessarily involves simplifying assumptions. The results of prior physical testing at loading rates consistent with walking [30], and the equivalence between short-time biphasic and incompressible elastic material responses [31, 32], justify treating articular cartilage as a linear elastic material in the present context. Subchondral bone was modeled as rigid, based on mechanical data showing that its compressive modulus is almost two orders of magnitude higher than that of articular cartilage [26, 33]. These seem acceptable approximations, at least for functional loads and loading rates. Prior physical validation of this DEA formulation [12] reinforces that these simplifications are reasonable. Other important assumptions made include the modeling of a single static loading condition. Differential gait alterations across the study population would likely influence the predictions of contact stress.

Elevated contact stress exposure is but one of several factors influencing a joint's propensity for OA development. To our knowledge, there exist no large-series data demonstrating the value of subject-specific contact stress as a predictor of incident symptomatic knee OA. Our prior work was novel in that it identified a correlation between maximum contact stress and risk for development of incident symptomatic tibiofemoral OA, albeit in a relatively small cohort of subjects. The capabilities of DEA were key to establishing that correlation, and those capabilities enable study of a much larger series of subjects than would be practicable by full tensorial stress analysis using techniques such as finite element or boundary element analysis.

Due to the extensive use of automation in model creation, alignment, and contact stress computation, procedures for verifying the quality of individual results must be considered. For the purposes of this study, results for each step were examined and confirmed visually, but this will not be feasible in contemplated studies involving thousands of modeling simulations. For this reason, robust and objective methods need to be developed to specifically identify poor solutions and return them to the analyst for further consideration.

## 4. Conclusion

The described methods provide a practical framework for utilizing information from large epidemiological and clinical studies to compute mean and peak articular contact stress exposures in the medial and lateral tibiofemoral compartments. The segmentation and registration steps of the DEA process appear to have good to excellent day-to-day and interrater reliability. This technique for estimating compartment-specific tibiofemoral contact stress may be useful for estimating biomechanical stress in large cohorts. In an ongoing study, the described methods have been used to analyze 200 knees from MOST subjects. Future addition of automated segmentation and quality control methods will significantly decrease investigator time investment and further improve the predictive value.

## Figures and Tables

**Table 1:** Segmentation reliability—contact stress intraclass correlation coefficients for day-to-day and inter-rater reliability (Shrout-Fleiss reliability single scores) for peak and mean contact stress in the medial and lateral compartments of the 10 knees studied.

Segmentation reliability	Compartment	Peak stress	Mean stress
Day-to-day	Medial	0.94	0.93
	Lateral	0.99	0.98

Interrater	Medial	0.87	0.84
	Lateral	0.96	0.95

**Table 2:** Registration reliability—contact stress intraclass correlation coefficients for day-to-day and inter-rater reliability (Shrout-Fleiss single score reliability) for peak and mean contact stress in the medial and lateral compartments of the 10 knees studied.

Registration reliability	Compartment	Peak stress	Mean stress
Day-to-day	Medial	0.93	0.94
	Lateral	0.95	0.96

Interrater	Medial	0.94	0.95
	Lateral	0.97	0.97
